# Automated external defibrillator location and socioeconomic deprivation in Great Britain

**DOI:** 10.1136/heartjnl-2023-322985

**Published:** 2023-08-28

**Authors:** Thomas Burgoine, David Austin, Jianhua Wu, Tom Quinn, Pam Shurmer, Chris P Gale, Chris Wilkinson

**Affiliations:** 1 MRC Epidemiology Unit, University of Cambridge School of Clinical Medicine, Cambridge, UK; 2 Academic Cardiovascular Unit, South Tees NHS Foundation Trust, Middlesbrough, UK; 3 Population Health Sciences Institute, Newcastle University, Newcastle upon Tyne, UK; 4 Wolfson Institute of Population Health, Queen Mary University of London, Barts and The London School of Medicine and Dentistry, London, UK; 5 Urgent and Emergency Health Care and Workforce Research Group, Kingston University, Kingston upon Thames, UK; 6 DS43 Community Defibrillators, Hartlepool, UK; 7 Leeds Institute of Cardiovascular and Metabolic Medicine, University of Leeds, Leeds, UK; 8 Department of Cardiology, Leeds Teaching Hospitals NHS Trust, Leeds, UK; 9 Hull York Medical School, University of York, York, UK

**Keywords:** PUBLIC HEALTH

## Abstract

**Objective:**

The early use of automated external defibrillators (AEDs) improves outcomes in out-of-hospital cardiac arrest (OHCA). We investigated AED access across Great Britain (GB) according to socioeconomic deprivation.

**Methods:**

Cross-sectional observational study using AED location data from The Circuit: the national defibrillator network led by the British Heart Foundation in partnership with the Association of Ambulance Chief Executives, Resuscitation Council UK and St John Ambulance. We calculated street network distances between all 1 677 466 postcodes in GB and the nearest AED and used a multilevel linear mixed regression model to investigate associations between the distances from each postcode to the nearest AED and Index of Multiple Deprivation, stratified by country and according to 24 hours 7 days a week (24/7) access.

**Results:**

78 425 AED locations were included. Across GB, the median distance from the centre of a postcode to an AED was 726 m (England: 739 m, Scotland: 743 m, Wales: 512 m). For 24/7 access AEDs, the median distances were further (991 m, 994 m, 570 m). In Wales, the average distance to the nearest AED and 24/7 AED was shorter for the most deprived communities. In England, the average distance to the nearest AED was also shorter in the most deprived areas. There was no association between deprivation and average distance to the nearest AED in Scotland. However, the distance to the nearest 24/7 AED was greater with increased deprivation in England and Scotland. On average, a 24/7 AED was in England and Scotland, respectively, 99.2 m and 317.1 m further away in the most deprived than least deprived communities.

**Conclusion:**

In England and Scotland, there are differences in distances to the nearest 24/7 accessible AED between the most and least deprived communities. Equitable access to ‘out-of-hours’ accessible AEDs may improve outcomes for people with OHCA.

**WHAT IS ALREADY KNOWN ON THIS TOPIC**

- Timely use of an automated external defibrillator (AED) is associated with improved outcomes in out-of-hospital cardiac arrest.
- Access to an AED may be different depending on the deprivation of a community, which may contribute to worse survival from cardiac arrest.

**WHAT THIS STUDY ADDS**

- This study shows that the distance to the nearest AED is shorter in England and Wales for more deprived communities, with no overall trend in Scotland.
- However, when considering AEDs that are accessible 24/7, the nearest device is further in more deprived neighbourhoods in England and Scotland, and nearer in Wales.

**HOW THIS STUDY MIGHT AFFECT RESEARCH, PRACTICE OR POLICY**

- Greater attention to equality of access to 24/7 AEDs has the potential to improve outcomes and save lives.

## Introduction

Automated external defibrillators (AEDs) can be used by untrained members of the public to provide rapid treatment of ventricular arrhythmias in out-of-hospital cardiac arrest (OHCA).^1^ Ambulances attend 30 000 people with OHCA each year in the UK.^2^ Up to 37% of OHCA are secondary to ventricular arrhythmia and may be effectively treated by AED prior to the arrival of an ambulance.^1–3^ The provision of AEDs in busy public places has been a policy priority in Great Britain (GB) since 1999,^4^ as early defibrillation is associated with improved survival in OHCA. With each minute of delay in defibrillation the chances of survival decrease by approximately 10%.^5 6^ Yet, AEDs are used in just one in ten patients with OHCA.^2^

Timely AED use depends on the time it takes to retrieve an AED, and therefore the distance from the OHCA to the nearest accessible AED. In the UK, publicly accessible AEDs are provided by organisations including schools, community groups and businesses (such as supermarkets). As a result, the distribution of AEDs and their hours of operation differ within and between communities, so that they are not always available ‘24/7’. Although OHCA rates are higher in more deprived neighbourhoods,^7^ this may not be matched by AED accessibility since more affluent areas typically have greater access to funds, health literacy and advocacy to obtain an AED for their community.^8^ This may contribute to inequalities in OHCA outcomes between socioeconomic groups.^9^

Previous research has identified that the density of AED provision is lower in more deprived communities in England.^9^ However, previous analyses have been limited by a lack of comprehensive data on AED location (as until recently it was not systematically collected), did not account for accessibility of the AED at different times of day, and relied on AED density as a proxy for the distance between an individual and their nearest AED.^9^ In 2022, the British Heart Foundation (BHF) collated locations of AEDs from ambulance services, individuals and organisations.^10^ In this study, we have used all postcodes in GB and the street location of registered AEDs to quantify the association between socioeconomic deprivation and the distance to AED location by country (England, Scotland, Wales) and according to AED hours of access.

## Methods

### AED data

Details about AEDs in GB are submitted to The Circuit by ambulance services, individuals, businesses and other organisations.^10^ Information about active AEDs in GB up to 14 October 2022 was provided by The Circuit, as well as information on accessibility (those accessible 24 hours per day, 7 days a week (24/7)) and location (latitude and longitude). Street locations for AEDs were mapped using a geographic information system (ArcGIS Pro, ESRI). We used June 2017 Ordnance Survey Code-Point Open with Polygons data to derive geographic centroids (hereafter referred to as ‘postcodes’) for all 1 677 466 postcodes across GB. Postcodes are the base unit of postal geography, and typically include around 15 addresses—although a large building (such as a tower block) may have more than one postcode.^11^

### Deprivation and rurality data

We overlaid country-specific deprivation and urban/rural status data, as these measures are not comparable between countries. We attributed 2019 lower super output area (LSOA) Index of Multiple Deprivation (IMD) and Welsh IMD deciles to postcodes in England and Wales, respectively. We attributed 2020 data zone Scottish IMD deciles to postcodes in Scotland. We also overlaid country-specific urban/rural status for LSOAs and data zones, using 2011 urban–rural classification data for England and Wales, and 2016 urban rural classification data for Scotland. There are seven domains of relative deprivation included in the English and Scottish IMD^12 13^ and eight in the Welsh IMD.^14^ These include measures of: income; employment; education, skills and training; health and disability; crime; barriers to housing and services; and living environment.

### Mapping

Using 2022 Ordnance Survey Highways road network data, we calculated the shortest street network distance in metres between each postcode and the nearest active AED, accessible 24/7 AED and time-restricted access AED, using ArcGIS Pro Network Analyst. The shortest street network distance was the sum of the Euclidean (straight line) distances between each of postcode and AED, to their closest points on the street network, and the distance along the street network between these two points.

### Statistical methods

We reported number of active defibrillators by country and type of AED. We summarised the distance (m) between each postcode and the nearest active AED using median (IQR) because distances were highly right skewed ((online supplemental figure 1), sktest all p<0.05). In the analysis below, distances were log-transformed prior to analysis and subsequently exponentiated.

10.1136/heartjnl-2023-322985.supp1 Supplementary data

We used linear mixed models to fit the log-transformed distances, using restricted maximum likelihood (REML) to provide unbiased estimates, to test the relationship between area deprivation and distance to each of the nearest active AED and AED accessible 24/7. We included a random intercept to account for a clustering effect at the LSOA level. Models were additionally adjusted for urban/rural status. We fitted each model stratified by country, as the construction of IMD varies by nation. Predicted marginal means and 95% CIs for deprivation deciles were predicted with model coefficients. We assessed whether the data violated the model assumptions such as non-linearity, heteroscedasticity and outliers using residual analysis and checked the normality assumption through quantile–quantile (Q–Q) plots. The use of REML allows for more efficient estimation of the random-effects parameters in the presence of fixed effects. Trends on deprivation score were calculated using a linear regression model, with estimated AED distance as dependent variable and significance defined at p trend <0.05. Analyses were conducted using Stata V.17 and R V.4.1.3.

The study was approved by The Circuit data governance board.

### Patient and public involvement

Our PPI co-author (PS) is one of the Trustees of DS43 Community Defibrillators, a charity she established with her husband Bill following the loss of their son, Danny, to cardiac arrest. She contributed to the interpretation of the research findings and will assist with dissemination of the findings and subsequent research.

## Results

In total, 78 425 unique AED locations were included in the analysis of which 55 576 (70.9%) were in England, 13 503 (17.2%) in Scotland and 9346 (11.9%) in Wales (table 1). Overall, 34 294 (43.7%) were accessible to the public 24/7 and 44 141 (57.3%) were available during restricted hours.

**Table 1:** Number of active defibrillators, overall (Great Britain) and by country

	All	24/7 (%)	Restricted (%)
Great Britain	78 425	34 294 (43.7)	44 141 (57.3)
England	55 576	25 632 (46.1)	29 944 (53.9)
Scotland	13 503	3054 (22.6)	10 449 (77.4)
Wales	9346	5608 (60.0)	3738 (40.0)

**Figure 1:**
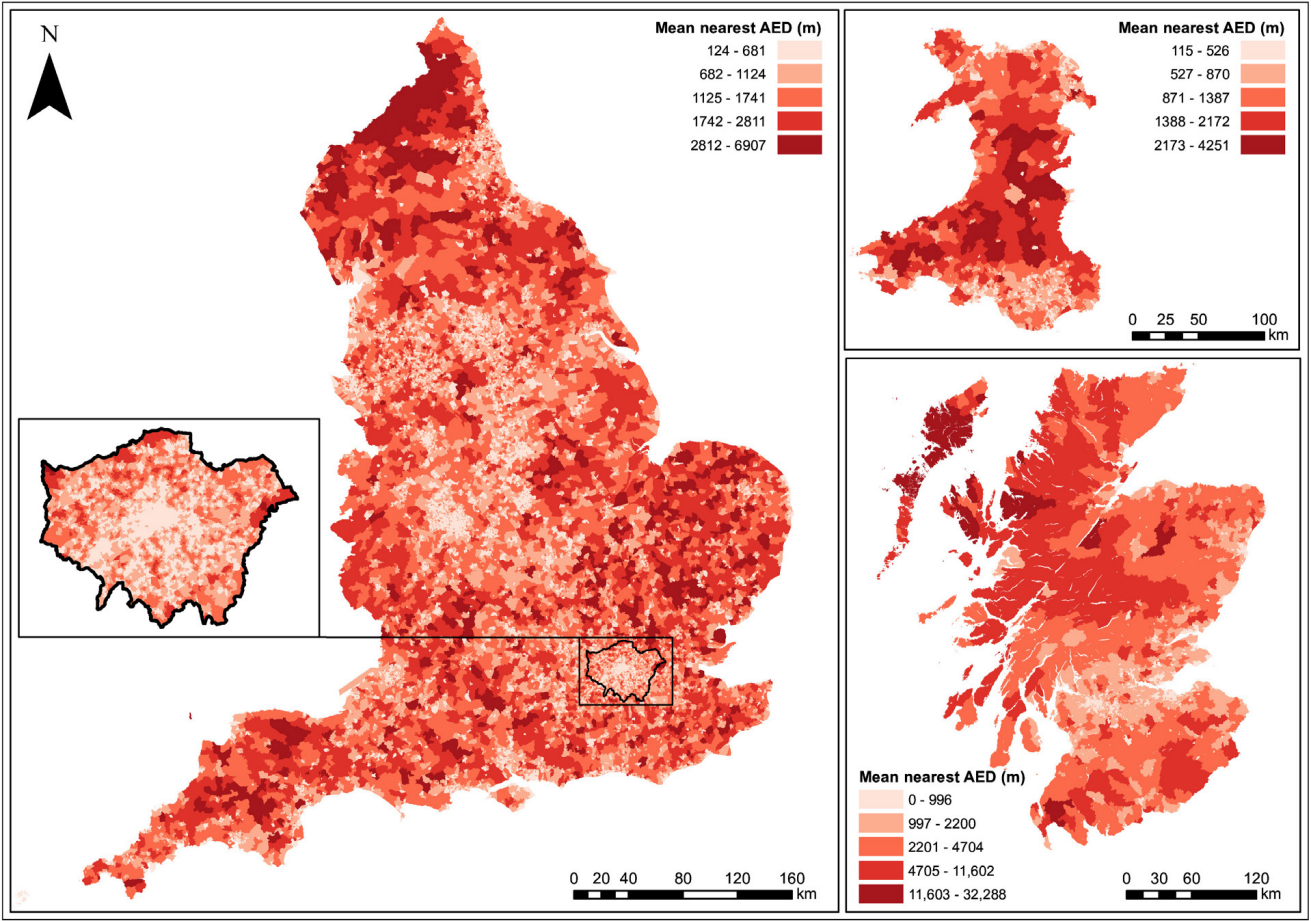
Mean street network distance (metres) to the nearest active automated external defibrillator (AED), across postcodes within lower super output areas (LSOAs) in England (Greater London inset), Scotland and WalesContains OS data © Crown copyright and database right (2023). Contains Royal Mail data © Royal Mail copyright and Database right (2023).

Across GB, the median distance from the centre of a postcode to the nearest active AED along the street network was 726.1 m (IQR 411–1221 m), ranging between 2.7 m and 49 km (table 2). The median distance was highest in Scotland (742.7 m), lowest in Wales (511.5 m) and was 738.8 m in England. The maximum distance to an AED was greatest in Scotland (49.1 km), followed by England (19.4 km) and was the shortest in Wales (14.9 km). Figure 1 shows the geographic distribution of mean distance (m) to the nearest active AED across postcodes within LSOAs, in England (Greater London inset), Scotland and Wales. When considering AEDs that were accessible 24/7, the median distances were higher. Across GB, the median distance to a 24/7 AED was 964.0 m, with a similar pattern of variation between the three nations (Scotland: 994.1 m; England: 990.8 m; Wales: 569.7 m). For restricted hours AEDs the median distance tended to be higher, with lower variation between the nations (1194.8 m in Scotland, 1230.4 m in England, 1174.2 m in Wales, table 2).

**Table 2:** Median street network distance (metres) to the nearest active automated external defibrillator

	Number of postcodes	Median (IQR), m	Range, m
Great Britain			
Any	1 671 943	726.1 (410.8–1221.0)	2.7–49 080.8
24/7	1 671 907	964.0 (524.7–1667.5)	4.2–49 206.4
Restricted	1 671 076	1224.4 (685.2–2294.6)	2.7–68 064.3
England			
Any	1 420 180	738.8 (422.6–1223.5)	2.7–19 439.9
24/7	1 420 160	990.8 (544.9–1683.3)	4.2–19 439.9
Restricted	1 420 149	1230.4 (693.8–2267.6)	2.7–45 141.0
Scotland			
Any	160 285	742.7 (407.2–1340.7)	4.5–49 080.8
24/7	160 274	994.1 (538.5–1832.3)	4.5–49 206.4
Restricted	159 459	1194.8 (646.0–2449.1)	5.5–68 064.3
Wales			
Any	91 478	511.5 (282.9–937.3)	5.5–14 936.8
24/7	91 473	569.7 (316.8–1033.4)	8.3–14 936.8
Restricted	91 468	1174.2 (625.8–2661.6)	5.5–24 634.8

### AED proximity and deprivation by country

#### England

**Figure 2:**
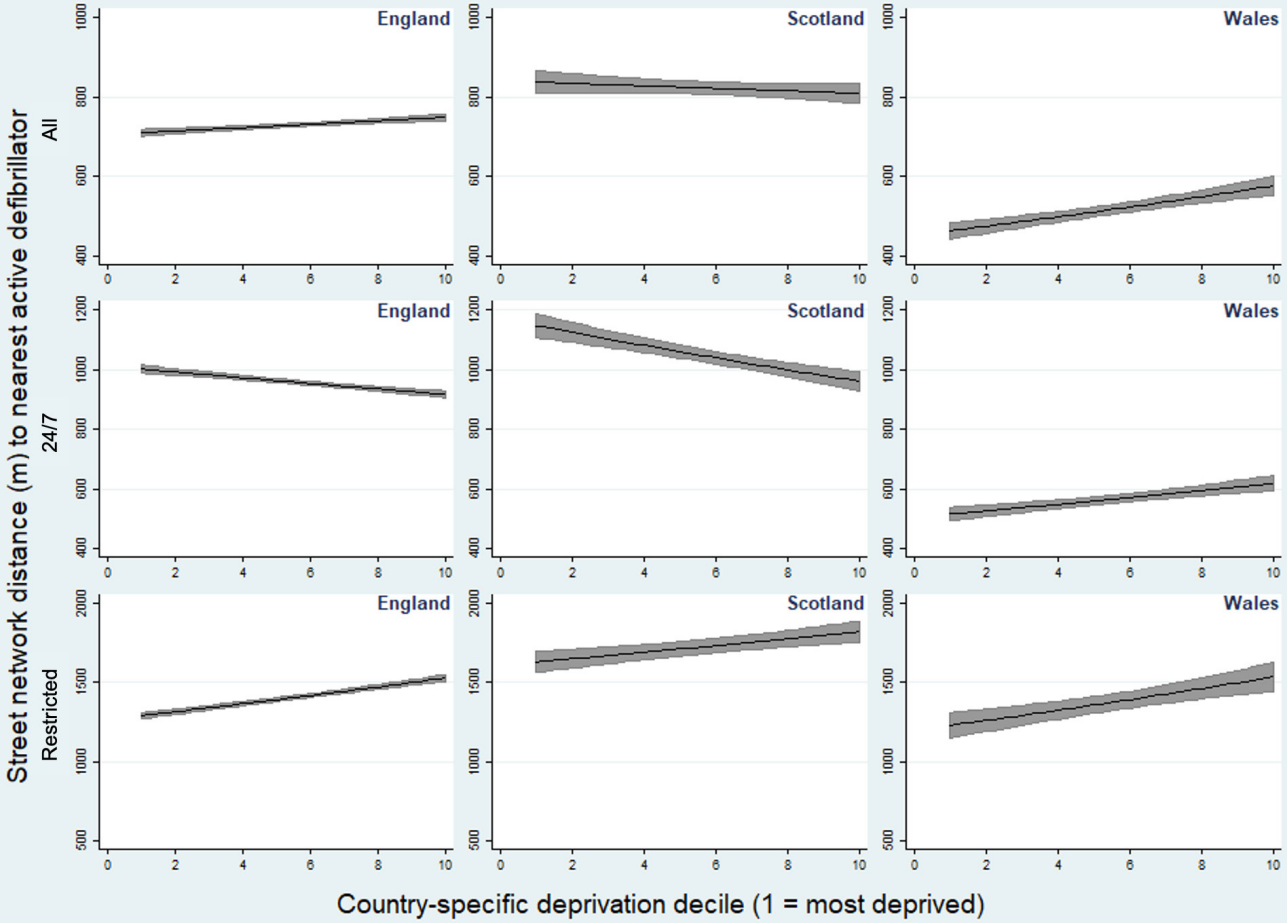
Adjusted predicted mean street network distance to nearest automated external defibrillator by country-specific deprivation decile. Metres (95% CI).

Increasing levels of deprivation were associated with lower distances to the nearest AED overall (p trend <0.05, figure 2). When hours of access were considered, there was an opposing trend. Increased deprivation was associated with an increased distance to the nearest 24/7 accessible AED (p trend <0.05), such that residents of the most deprived areas of England were on average 99.2 m further away from their nearest AED than those in the least deprived areas (D1: 1000.3 m, 95% CI 977.8 to 1022.8 vs D10: 901.1 m, 881.2 to 920.9; table 3). In contrast, the average distance to a restricted hours AED was highest in the least deprived deciles (D9: 1499.9 m, 95% CI 1465.1 to 1534.8; D10: 1498.3 m, 1463.2 to 1533.5) and lowest in the most deprived deciles (D1: 1306.2, 95% CI 1274.2 to 1338.2; D2: 1302.4, 1270.7 to 1334.1; p trend<0.05).

**Table 3:** Adjusted predicted mean street network distance to nearest automated external defibrillator by country-specific deprivation decile

	Deprivation decile (1=most deprived)									
	1	2	3	4	5	6	7	8	9	10
England (m)										
Any	720.8	715.9	714.9	706.5	724.8	731.3	735.2	744.2	756.0	741.0
	(706.4 to 735.3)	(701.6 to 730.2)	(700.7 to 729.1)	(692.7 to 720.4)	(710.7 to 739.0)	(717.0 to 745.5)	(720.9 to 749.6)	(729.7 to 758.7)	(741.3 to 770.8)	(726.5 to 755.3)
24/7	1000.3	1006.6	970.9	954.5	969.8	961.5	947.0	931.8	946.4	901.1
	(977.8 to 1022.8)	(984.0 to 1029.1)	(949.3 to 992.6)	(933.4 to 975.6)	(948.5 to 991.1)	(940.4 to 982.7)	(926.2 to 967.7)	(911.4 to 952.3)	(925.6 to 967.1)	(881.2 to 920.9)
Restricted	1306.2	1302.4	1329.9	1339.0	1389.5	1434.9	1455.8	1492.8	1499.9	1498.3
	(1274.2 to 1338.2)	(1270.7 to 1334.1)	(1297.9 to 1361.9)	(1307.4 to 1370.6)	(1357.2 to 1421.9)	(1401.7 to 1468.1)	(1422.1 to 1489.4)	(1458.1 to 1527.4)	(1465.1 to 1534.8)	(1463.2 to 1533.5)
Scotland (m)										
Any	873.4	841.7	797.6	757.8	824.0	848.3	820.5	837.0	851.1	765.6
	(824.6 to 922.2)	(795.0 to 888.4)	(753.6 to 841.7)	(716.7 to 798.9)	(780.0 to 867.9)	(803.5 to 893.1)	(777.2 to 863.8)	(792.5 to 881.4)	(804.6 to 897.6)	(722.9 to 808.3)
24/7	1221.1	1119.3	1071.8	999.1	1018.0	1058.7	1038.3	1040.2	1037.9	904.0
	(1149.8 to 1292.4)	(1054.3 to 1184.2)	(1009.9 to 1133.8)	(942.3 to 1055.9)	(961.0 to 1075.0)	(1000.0 to 1117.6)	(980.6 to 1096.1)	(982.2 to 1098.2)	(978.6 to 1097.2)	(851.3 to 956.8)
Restricted	1581.6	1662.0	1610.5	1568.9	1817.6	1814.4	1717.1	1756.0	1926.4	1642.8
	(1481.2 to 1681.9)	(1557.2 to 1766.7)	(1509.7 to 1711.3)	(1472.9 to 1664.8)	(1709.1 to 1926.0)	(1709.5 to 1919.3)	(1617.9 to 1816.3)	(1653.3 to 1858.7)	(1808.2 to 2044.7)	(1538.8 to 1746.8)
Wales										
Any	490.9	459.5	470.7	452.6	541.8	509.5	596.7	544.6	526.7	585.9
	(455.0 to 526.7)	(426.3 to 492.7)	(437.1 to 504.2)	(420.8 to 484.7)	(504.1 to 579.6)	(474.2 to 544.8)	(555.4 to 638.0)	(506.8 to 582.4)	(489.7 to 563.7)	(543.7 to 628.1)
24/7	559.9	513.3	510.9	491.5	586.2	549.2	654.9	600.4	578.2	621.4
	(518.4 to 601.4)	(475.6 to 550.9)	(473.9 to 547.9)	(456.2 to 526.8)	(544.6 to 627.8)	(510.5 to 587.9)	(608.7 to 701.0)	(557.9 to 642.9)	(536.9 to 619.5)	(575.9 to 666.8)
Restricted	1183.6	1228.8	1237.0	1338.1	1479.8	1409.0	1537.9	1388.1	1380.5	1539.4
	(1059.5 to 1307.7)	(1101.0 to 1356.6)	(1110.7 to 1363.3)	(1202.7 to 1473.4)	(1332.9 to 1626.7)	(1270.6 to 1547.5)	(1387.4 to 1688.5)	(1251.5 to 1524.6)	(1242.2 to 1518.8)	(1380.4 to 1698.3)

Multi-level model accounting for lower super output area level clustering; adjusted for urban/rural status. Metres (95% CI).

#### Scotland

In Scotland, there was no statistically significant trend in overall AED provision by deprivation (p trend=0.205), although the predicted mean distance to the nearest AED was on average 108 m further for the most compared with the least deprived decile (D1 873.4 m, 95% CI 824.6 to 922.2 vs D10 765.6 m, 722.9 to 808.3; table 3). As in England, there was a statistically significant trend in opposing directions when hours of access were considered, such that the distances to the nearest AED were further for more deprived neighbourhoods for 24/7 access and closer for restricted access AEDs (p trend <0.05 for each, figure 2).

#### Wales

There was an association between increased deprivation and decreased distance to the nearest AED overall (p trend <0.05), such that postcodes in the most deprived decile were on average 95 m closer than the least deprived (490.9 m, 95% CI 455.0 to 526.7 vs 585.9 m, 543.7 to 628.1). The same association was observed for 24/7 accessible and restricted access AEDs, with statistically significant evidence of trend (p trend<0.05 for each, figure 2).

## Discussion

Across all postcodes in GB, the distance to an AED varied between 3 m and 49 km, with the greatest median distance in Scotland (743 m) and lowest in Wales (512 m). In England, more deprived postcodes tended to have shorter distances to the nearest active AED. The same trend was not observed for Scotland, however, the average distance to the nearest AED was 108 m further for postcodes in the most compared with least deprived postcodes. In England and Scotland, more deprived postcodes tended to have a longer distance to the nearest 24/7 AED. In Wales, more deprived neighbourhoods had a shorter distance to any active AED, 24/7 accessible AED, and restricted hours AED than the least deprived.

To the best of our knowledge, this is the largest and most comprehensive published evaluation of AED access in GB. We make use of the latest and most accurate data on AED locations. For the first time, we were able to calculate the distance from the centre of over 1.6 million postcodes to the nearest AED and also account for the accessibility of AEDs ‘out of hours’. However, we recognise the limitations of our work. Registration of AEDs is not mandatory, so while this study uses the most comprehensive dataset available, it is likely to be incomplete. The distance calculations made use of road network only, so it is possible that shorter walking routes exist to the nearest AED than those that we were able to calculate. We also recognise that there are important reasons why AEDs may not be distributed according to population proximity alone. Population density is dynamic as people move between locations for work and recreation. Places where people congregate sporadically (eg, community halls, stadia and places of worship) may have a very high population density at some times but not others. The average baseline risk of cardiac arrest varies within and between populations, and places where people exercise such as gyms and sports centres may have a higher incidence of cardiac arrest than an average postcode. Indeed, recent work has shown that about a third of all OHCAs occur within 300 m of a school, and therefore suggested a strategy of placing AEDs in schools.^15^ These considerations are likely to explain some of the heterogeneity in AED placement.

There is clear evidence that timely defibrillation is associated with improved outcomes in cardiac arrest.^5 6 16^ A public access AED is likely to be available for use more rapidly than an ambulance would be able to attend, particularly given recent pressures on ambulance services. The walking distance to the nearest accessible AED is therefore an integral component in the chain of survival for OHCA,^17^ and inequalities in access are likely to have a significant impact on patient outcomes. In England and Scotland, those most deprived had consistently poorer access to an AED accessible 24/7, which is important as 29% of OHCAs occur at weekends and 40% are between 18:00 and 06:00 in England.^18^ At a typical walking speed of 1.3 m/sec and based on a round trip to a 24/7 accessible AED,^19^ we observed that those in the most deprived areas would be delayed access to an AED by 2½ and 8 min, respectively, in England and Scotland, when compared with those in the least deprived areas. Speculatively, this may be due to the location of AEDs within public buildings or supermarkets without 24/7 accessibility. These amenities may be located in areas with lower average house prices, although this relationship is not straightforward.^20^ Regardless of time of day, access to an AED would be delayed by nearly 3 min if the OHCA occurred in one of Scotland’s most deprived 10% of areas.

Previous work showed that the most deprived communities in New Zealand had the lowest availability of public access defibrillators.^21^ In Berlin, public defibrillator access was lowest in districts with below median income.^22^ A previous study in England also examined the characteristics of small areas with and without AED access and found that AEDs were more numerous in affluent areas.^9^ Our study provides evidence of a more nuanced pattern, with differing trends by hours of access and by country. In Scotland, the opposing trends for 24/7 and restricted access AEDs led to a combined non-significant association. Additionally, we had the benefit of a more comprehensive dataset (the newly available The Circuit) and calculated street network distance to the nearest AED from all postcodes, which is a more meaningful measure of access than area-level density.

Other studies have suggested a modelling approach to determining AED location based on previous OHCA incidence,^23 24^ although this may not predict future OHCA frequency in a location. Community groups are increasingly providing AEDs,^25^ and in deciding where to optimally site these there is the potential to use a location allocation model based on postcode data, walking time to the nearest AED and weighted population risk in each postcode. This approach has been successfully used to plan the location of antenatal classes and ambulance stations for populations.^26 27^ Additionally, future housing developments should consider AED access as part of the planning process. In the future, AED delivery by drone may be beneficial, particularly in rural settings.^28^

There are many factors that impact on survival following OHCA, which may vary by socioeconomic status, including provision (and potentially quality) of cardiopulmonary resuscitation. Previous work has identified a lack of awareness of AEDs, alongside a reluctance and limited confidence in using AEDs even where they are available.^29^ Indeed, an analysis of the Danish Cardiac Arrest Registry found that between 2001 and 2014, bystander defibrillation was provided in just 2.4% of cases of OHCA—with higher rates among patients with greater income.^30^ There is a need for increased public engagement with, and education on the importance of prompt CPR, and how to retrieve and use an AED. This could can be supported through the use of GPS-enabled smartphone apps, for example.

## Conclusion

Across GB, the median distance to an AED was highest for postcodes in Scotland and lowest for those in Wales. In England but not Scotland, more deprived areas tended to have shorter distances to their nearest active AED. The same was true for Wales, with distances to nearest 24/7 accessible AEDs also tending to be shorter in more deprived areas. In England and Scotland, those in the most deprived areas had to travel over 1 km to their nearest 24/7 accessible AED, which tended to be further away than in less deprived areas. More equitable future AED placement has the potential to save lives and improve neurological outcomes for people with OHCA, as does extending the hours that existing AEDs are accessible to members of the public.

## Data Availability

Data are available upon reasonable request. Data may be obtained from a third party and are not publicly available. Data are available by application to The Circuit.
