# Supplementary material for: Automated external defibrillator location and socioeconomic deprivation in Great Britain
Source: Heart. 2023 Aug 28;110(3):188–94. doi: 10.1136/heartjnl-2023-322985 (PMC10850630; doi:10.1136/heartjnl-2023-322985)
Supplement: Supplementary data [file heartjnl-2023-322985supp001.pdf]

Supplementary appendix

Supplementary table 1: Unadjusted mean street network distance to nearest automated external defibrillator (AED) by country-specific deprivation decile. Metres (95% confidence interval).

	Deprivation decile (1 = most deprived)									
	1	2	3	4	5	6	7	8	9	10
**England**										
Any	671.3	671.4	676.4	683.2	714.8	729.5	733.2	737.4	748.2	723.3
	(658.0,684.6)	(658.1,684.7)	(663.0,689.8)	(669.7,696.7)	(700.7,728.9)	(715.1,743.9)	(718.7,747.6)	(722.8,751.9)	(733.4,763.0)	(709.0,737.6)
24/7	987.8	995.3	961.8	949.4	968.2	962.2	947.5	931.2	945.5	897.9
	(966.1,1009.5)	(973.4,1017.2)	(940.6,982.9)	(928.5,970.3)	(947.0,989.5)	(941.0,983.3)	(926.7,968.3)	(910.8,951.7)	(924.7,966.3)	(878.2,917.6)
Restricted	945.5	966.3	1017.5	1104.7	1228.0	1320.2	1337.8	1341.8	1342.7	1278.5
	(921.1,969.9)	(941.4,991.2)	(991.3,1043.8)	(1076.3,1133.2)	(1196.4,1259.7)	(1286.2,1354.2)	(1303.3,1372.2)	(1307.2,1376.4)	(1308.1,1377.4)	(1245.6,1311.5)
**Scotland**										
Any	720.7	705.1	676.5	676	785.6	892.3	849.3	830.4	749.7	633.8
	(680.5,760.9)	(665.8,744.4)	(638.8,714.1)	(638.4,713.7)	(741.9,829.3)	(842.7,941.8)	(802.2,896.5)	(784.1,876.6)	(707.9,791.6)	(598.5,669.2)
24/7	1046.9	971.7	940.1	914.0	984.3	1110.9	1075.1	1039.6	939.5	777.2
	(986.6,1107.2)	(915.8,1027.7)	(886.0,994.3)	(861.4,966.6)	(927.7,1040.9)	(1047.1,1174.7)	(1013.3,1136.8)	(979.7,1099.4)	(885.3,993.7)	(732.4,822.0)
Restricted	958.1	1036.7	1030.2	1107.7	1466.3	1799.5	1655.2	1552.7	1333.7	1001.0
	(893.9,1022.3)	(967.2,1106.1)	(961.2,1099.3)	(1033.5,1181.9)	(1367.8,1564.9)	(1678.9,1920.2)	(1544.6,1765.8)	(1448.9,1656.5)	(1244.4,1423.1)	(934.1,1068.0)
**Wales**										
Any	430.0	407.0	430.2	421.1	526.2	511.3	609.3	545.9	500.5	527.6
	(398.2,461.8)	(377.0,437.0)	(398.6,461.9)	(390.2,452.0)	(487.6,564.7)	(474.0,548.6)	(564.8,653.8)	(505.9,585.9)	(463.7,537.3)	(488.5,566.6)
24/7	504.6	466.8	476.5	464.9	574.1	552.2	667.7	603.1	556.4	572.6
	(467.3,541.9)	(432.4,501.1)	(441.5,511.5)	(430.8,499.0)	(532.0,616.1)	(511.9,592.5)	(619.0,716.5)	(558.9,647.3)	(515.6,597.3)	(530.3,614.9)
Restricted	796.8	851.4	926.0	1046.1	1286.7	1330.0	1515.9	1304.4	1138.4	1109.9
	(706.1,887.5)	(754.6,948.2)	(820.7,1031.2)	(927.3,1165.0)	(1140.6,1432.8)	(1179.1,1480.9)	(1343.9,1688.0)	(1156.3,1452.6)	(1009.0,1267.7)	(983.4,1236.5)

Multi-level model accounting for lower super output area level clustering

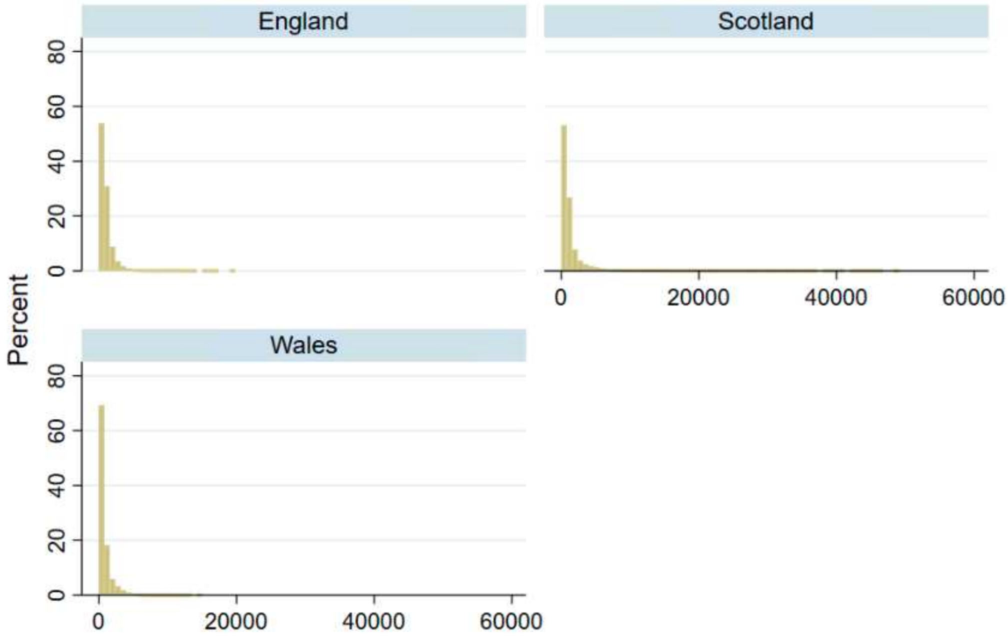

Supplementary figure 1A: Total distance (m) to the nearest automated external defibrillator

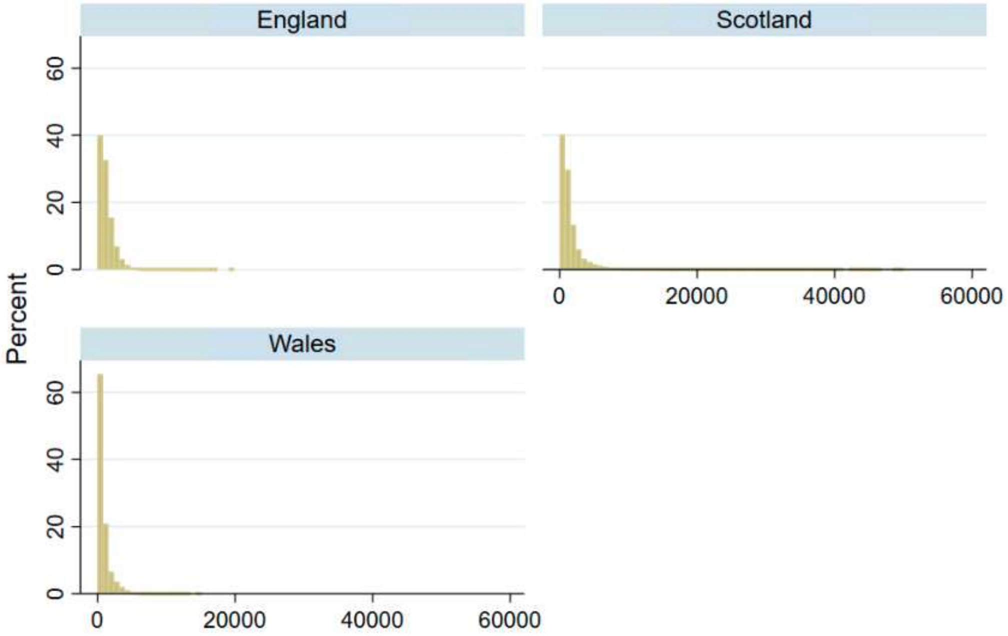

Supplementary figure 1B: Total distance (m) to the nearest 27/7 access automated external defibrillator
